# Roles of TGF-β1 in Viral Infection during Pregnancy: Research Update and Perspectives

**DOI:** 10.3390/ijms24076489

**Published:** 2023-03-30

**Authors:** Quang Duy Trinh, Ngan Thi Kim Pham, Kazuhide Takada, Hiroshi Ushijima, Shihoko Komine-Aizawa, Satoshi Hayakawa

**Affiliations:** Division of Microbiology, Department of Pathology and Microbiology, Nihon University School of Medicine, Tokyo 173-8610, Japan

**Keywords:** TGF-β1, transforming growth factor, virus, pregnancy, ToRCH, Zika, influenza, rubella, HIV, SARS-CoV-2

## Abstract

Transforming growth factor-beta 1 (TGF-β1) is a pleiotropic growth factor playing various roles in the human body including cell growth and development. More functions of TGF-β1 have been discovered, especially its roles in viral infection. TGF-β1 is abundant at the maternal–fetal interface during pregnancy and plays an important function in immune tolerance, an essential key factor for pregnancy success. It plays some critical roles in viral infection in pregnancy, such as its effects on the infection and replication of human cytomegalovirus in syncytiotrophoblasts. Interestingly, its role in the enhancement of Zika virus (ZIKV) infection and replication in first-trimester trophoblasts has recently been reported. The above up-to-date findings have opened one of the promising approaches to studying the mechanisms of viral infection during pregnancy with links to corresponding congenital syndromes. In this article, we review our current and recent advances in understanding the roles of TGF-β1 in viral infection. Our discussion focuses on viral infection during pregnancy, especially in the first trimester. We highlight the mutual roles of viral infection and TGF-β1 in specific contexts and possible functions of the Smad pathway in viral infection, with a special note on ZIKV infection. In addition, we discuss promising approaches to performing further studies on this topic.

## 1. Introduction

The human body has to experience significant life changes, including pregnancy status for women. Pregnancy is a particular period not only recognized by various physical changes but also includes immune tolerance, an essential establishment of the immune system to avoid a rejection of the mother against the fetus. The placenta has an indispensable role as a barrier to protect the fetus from any possible vertical infections from the mother, addressing a balance between the tolerance of an allogeneic fetus and the protection against pathogens naturally established at the maternal–fetal interface in a healthy pregnancy [1,2]. With the placenta’s notable physical and immunological roles, pathogens, such as viruses, cannot be easily transmitted to the fetus. However, by utilizing unknown mechanisms, some viruses can still pass through the placenta in some cases [3,4,5,6,7,8].

Viral infection during pregnancy can result in congenital viral syndromes such as congenital rubella syndrome (CRS) or congenital Zika syndrome (CZS), particularly in the first trimester. CRS can occur throughout the pregnancy; however, 90% of the cases of CRS were reported as a result of a rubella virus (RuV) infection in the first trimester [9]. For Zika virus (ZIKV) infection, it is known that the risk of a structural birth defect among infants born to mothers with ZIKV infection during pregnancy ranges from 5 to 10%, with higher incidences when the infection occurs in the first trimester [10,11]. However, the mechanisms for which these viral infections affect trophoblasts, a natural barrier to prevent the fetus from being infected due to a virally infected mother, are not well understood. Trophoblasts are generally well-known for their resistance or low susceptibility to various viruses [12,13].

In a healthy pregnancy, the first trimester is characterized by a balance between the invasion of trophoblast cells and fetal-maternal immune tolerance, with the predominant role of regulatory T cells (Treg cells) being indispensably regulated by TGF-β1 [14,15,16]. TGF-β1 is a pleiotropic growth factor with various functions in cell growth and differentiation leading to the development of the human body. During pregnancy, TGF-β1 plays essential roles in cell growth and differentiation, trophoblast cell invasion, maintenance of fetal-maternal immune tolerance, and uterine spiral artery remodeling [17,18,19].

TGF-β1 also plays some roles in viral infection [20,21,22,23,24,25,26,27,28,29,30,31,32,33]. It is worthy of note that it can promote ZIKV infection in the immortalized first-trimester trophoblast cells via the Smad pathway [34]. This finding provides a potential approach to studying the mechanisms of transplacental viral infections and congenital viral syndromes. To perform more studies to understand further the role of TGF-β1 in ZIKV infection in trophoblasts and in other viral infections at the maternal–fetal interface, this review was conducted to give a comprehensive picture of the current understanding of its roles in viral infection during pregnancy. We briefly focus on the infection of the viruses of the typical ToRCH pathogens and other known potentially transplacental transmission or recently emerging viruses such as human immunodeficiency virus (HIV), influenza A virus (IAV), hepatitis B virus (HBV), ZIKV, and severe acute respiratory syndrome coronavirus 2 (SARS-CoV-2). The pathogenesis of typical ToRCH pathogens (refers to toxoplasma, others, RuV, cytomegalovirus (CMV), and herpes simplex virus (HSV)), and ZIKV, which is included in the suggested newest ToRCHZ complex [35,36,37], as well as the above-mentioned other viruses causing vertical transmission, has been reviewed in detail elsewhere [38,39,40,41]. In this review, we also highlight the Smad pathway and its effect on ZIKV binding and replication in trophoblast cells. In addition, promising approaches to performing further studies into this topic have also been discussed.

Method:

Besides general information related to pregnancy and placenta, to search for available literature suitable for the title and content of this review, the following keywords were used in PubMed: “TGF-β1” or “TGFbeta1” or “transforming growth factor beta 1”, “virus”, “infection”, and “pregnancy”. 

Literature about the roles of TGF-β1 in various viral infections was searched using the keyword “TGF” and one of the following virus names: “HIV”, “influenza”, “cytomegalovirus” or “CMV”, “HBV”, “rubella”, “Zika”, “HSV”, “SARS-CoV-2”. Other keywords, such as “trophoblast” or “Hofbauer,” were added to more specific topics.

For information about TGF-β1, the following keywords were used: “TGF-β1” or “transforming growth factor beta 1”, “function,” “pathway”, “pregnancy”, “placenta”, “first trimester”. Articles pertinent to this review topic were selected for discussion.

## 2. Trophoblasts and the Placenta Villi in Pregnancy

During pregnancy, the placenta is not only a fetal organ responsible for nutrient and gas exchange between the mother and the fetus but also a barrier to protecting the fetus against various infectious pathogens, including viruses. This review focuses on the development of trophoblasts and the placenta in the first trimester, the critical period for placental formation and fetal organogenesis.

Regarding placental formation, in the first trimester, trophoblasts, the first structure of the placenta, arise from the trophectoderm of the blastocyte, taking place at the end of the first week of conception. With a branching villous structure, the placenta contains many villi that can be divided into two types: anchoring and floating villi. Each villus contains an outer bilayer of trophoblasts functioning in nutrient exchanges and an inner core containing placental blood vessels which link to fetal circulation. The outer trophoblasts comprise two subpopulations, an outer layer of continuous multinucleated syncytiotrophoblasts (STBs) and an inner layer of mononuclear cytotrophoblasts (CTBs), which can fuse into the outer layer. For anchoring villi, the CTBs of the villous tips differentiate into extravillous trophoblasts (EVTs) that migrate out and invade into the decidua. The EVTs not only attach the placenta, anchoring it to the uterus wall, but they also invade the maternal spiral arteries and transform them into wide vessels capable of supplying significant and constant maternal blood to the fetus [2,42,43,44]. Therefore, CTBs, and especially the EVTs, have a chance to come in contact with the mother’s blood since the first trimester, while the STBs are directly exposed to maternal blood from the second trimester of pregnancy onwards. Consequently, trophoblast cells are considered the first barrier in protecting the fetus from potential infections originating from the mother’s side. For viral infection in pregnancy which results in high incident rates of a congenital viral syndrome such as RuV, HCMV, and ZIKV, research into these viral infections of first-trimester trophoblasts is considered key to opening the gate to their transplacental transmission mechanisms.

## 3. Viral Infections in Pregnancy

The current pandemic of SARS-CoV-2 infection has raised concerns about unfavorable impacts on maternal and fetal health, particularly in light of recent outbreaks of emerging viruses such as ZIKV. Infections in pregnancy can cause various adverse pregnancy outcomes, including premature labor, pregnancy loss, and stillbirth. In addition, once vertical transmission occurs, it is one of the significant causes of morbidity and mortality in pregnancy, leading to severe diseases in the fetus, including birth defects and congenital infection [4,45,46,47] (Table 1).

**Figure 1:**
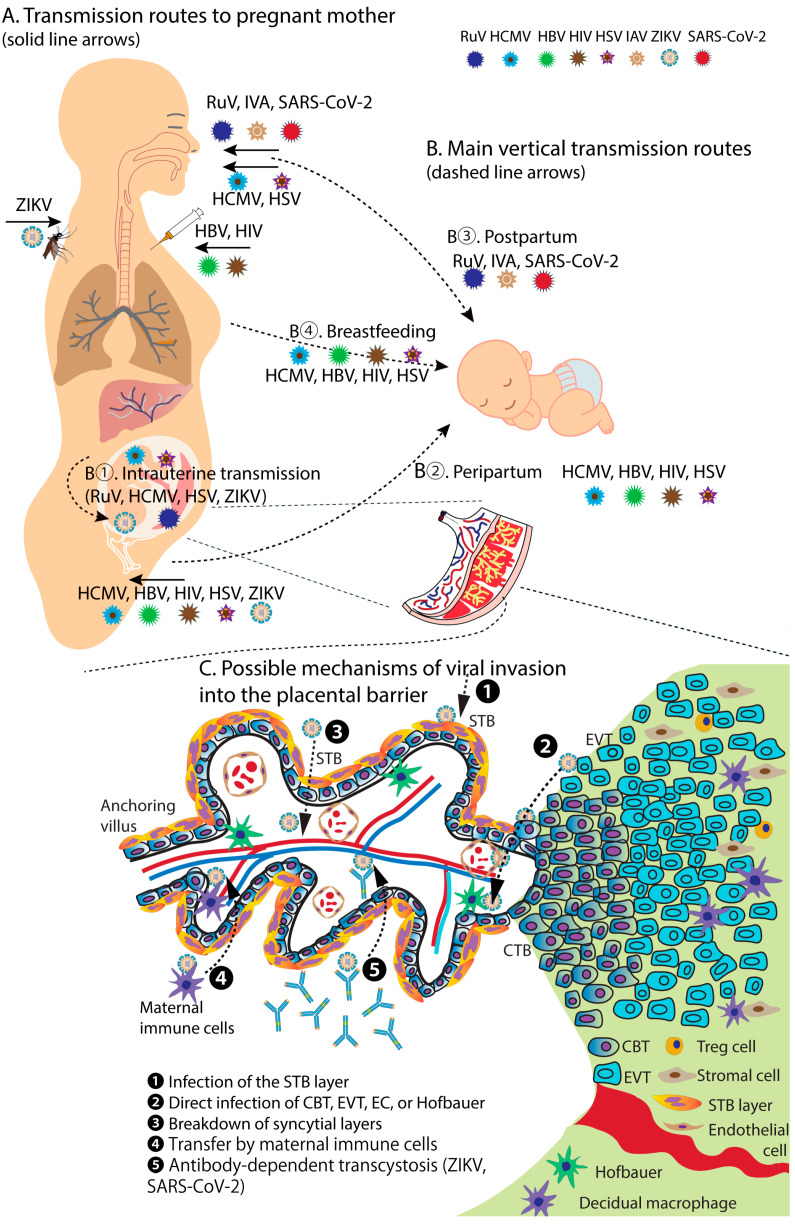
Viral transmission to pregnant mother and her fetus/baby. (**A**) Transmission routes to pregnant mother. (**B**) Vertical transmission routes. (**C**) Intrauterine transmission-possible mechanisms of virus invasion into the placenta barrier. Abbreviations: RuV, rubella virus; HCMV, human cytomegalovirus; HIV, human immunodeficiency virus; HBV, hepatitis B virus; HSV, herpes simplex virus; IAV, influenza A virus; ZIKV, Zika virus; SARS-CoV-2, severe acute respiratory syndrome coronavirus 2; CBT, cytotrophoblast; STB, syncytiotrophoblast; EVT, extravillous trophoblast; EC, endothelial cells; T reg, regulatory T cells.

A healthy pregnancy is a tightly regulated phenomenon derived from the interconnection between the mother and the fetus. During pregnancy, immune cells from the maternal and fetal compartments interact to promote a tolerogenic milieu suitable for fetal development, providing adequate defense against pathogens. Infectious agents, especially ToRCHZ and other viral causative agents, at the maternal–fetal interface are associated with adverse pregnancy outcomes and fetal loss [86,87,88,89]. Mother-to-child transmission (MTCT) of viruses can occur through multiple routes, including direct transplacental infection with placental damage or disruption of the maternal–fetal barrier (such as CMV, RuV, ZIKV, or HIV), ascending transmission from the vaginal cervical area (HSV), transplacental immune transfer of maternal antibodies which enhances viral infection (ZIKV), perinatal transmission (HIV, HBV), postnatal transmission through breastfeeding (HCMV, HIV, HBV, HSV) [55,58,64,76,90,91,92] (Figure 1 and Table 2).

### 3.1. RuV

This virus belongs to the genus *Rubivirus* in the family *Matonaviridae* [111]. It often causes systemic infection in children and young adults with a clinically mild, self-limited illness with fever and a generalized erythematous maculopapular rash. However, RuV is a well-known virus in reproductive health, with a concern for potentially causing dire consequences for the fetus, although the mechanism for maternal–fetal transmission is not well established. Infection with RuV during pregnancy, especially if the infection occurs in early pregnancy, can result in various adverse pregnancy outcomes such as miscarriage, fetal death, stillbirth, or infants born with congenital disabilities including cataracts, sensorineural hearing loss, psychomotor or mental retardation, known as the CRS [48,49,112,113]. Vaccination against RuV has been available for decades, and the eradication of rubella has reached approximately half the total number of countries in the world [48]. RuV infection is still a concern as its epidemics and CRS have still occurred, leading to the recently reinforced RuV vaccination in some regions [50,114,115,116]. It is well established that for most of the CRS cases, the infection and vertical transmission occurred in the first trimester [9]. However, in vitro studies showed that RuV has low infectivity in trophoblasts and suggest that some factors may affect the infection in the first trimester [8,13,92,117].

### 3.2. Human Cytomegalovirus

Human cytomegalovirus (HCMV) is one member of the herpesvirus family that establishes a lifelong latency following primary infection. It is yet an under-recognized infectious cause of newborn malformation, although endemic worldwide. Over 50% of the world’s population is estimated to be infected with HCMV [118,119,120]. The mother infected with HCMV in pregnancy can transmit the virus to the fetus, causing the congenital cytomegalovirus infection, either asymptomatic or with clinical manifestations. These adverse outcomes include jaundice, hepatosplenomegaly, microcephaly, intrauterine growth restriction, psychomotor and sensorineural disabilities including hearing and vision loss, or death [53,54,55]. In developed countries, congenital cytomegalovirus infection has become the most prevalent infection-related cause of congenital neurological defects since the introduction of the universal rubella vaccination. Vertical HCMV transmission can occur during intrapartum or breastfeeding [121,122]. However, intrauterine transmission through the transplacental crossing of the virus is essential as this infection route results in greater incidences of sequelae compared with intrapartum and postnatal transmission [123,124,125]. In vitro studies reported that cytotrophoblasts were permissive to HCMV replication. Villous syncytiotrophoblasts could be permissively infected by HCMV; however, the infection required high virus titers, and the progeny virus remained predominantly cell-associated [123,126,127].

### 3.3. HIV

Infection with HIV results in defective cellular immunity and opportunistic infections [59]. HIV can be transmitted from a mother to her child at any time in pregnancy, during the intrauterine, intrapartum, or breastfeeding periods. However, most infants are infected during delivery. There are some factors increasing the MTCT risk, such as the absence of antiretroviral treatment during pregnancy, vaginal delivery, breastfeeding, maternal seroconversion during pregnancy or breastfeeding, high maternal plasma viral RNA load during pregnancy, and advanced maternal HIV disease [57,128]. In the absence of intervention, the rate of transmission of HIV from a mother living with HIV to her child ranges from 15% to over 40% [58,61]. With the inclusion of antiretroviral drugs during pregnancy and the choice of delivery route, the transmission rate amounted to less than 2% or even decreased to almost zero in some settings, including free access to antiretroviral therapy [60,96,129,130,131,132]. Trophoblasts are unlikely to be infected with HIV or with low viral production, and vertical transmission is thought to be through CD4+ endothelial tissues or CD4+ Hofbauer cells [98,133,134,135,136].

### 3.4. HBV

The Hepatitis B virus (HBV) is well-known for causing chronic hepatitis, potentially leading to cirrhosis or liver cancer. In pregnancy, HBV infection has been associated with the risk of adverse maternal and infant outcomes in a highly endemic setting but not associated with adverse pregnancy outcomes in a low-burden setting such as in the US [65,99]. Vertical transmission to newborn infants of HBV was reported and was positively correlated with the high viral load of pregnant women, especially in the third trimester [100]. It was reported that up to 85% of infants born to HBeAg-positive mothers developed chronic HBV infection [137,138]. It raises concern about the possibility of MTCT of HBV in the fetus during pregnancy and the infants’ chronic infection state in early life thereafter.

Intrauterine transmission of HBV was noted in some studies [101,139,140,141]. HBV is not cytopathogenic, and there is no evidence of placental damage caused by HBV. The findings that acute hepatitis B occurring in the first or second trimester of pregnancy rarely caused HBV infection in infants as well as the lack of anti-HBc IgM in newborn infants of HBV-infected mothers, indicating that the virus does not easily cross the placenta [139,140], suggesting the existence of a placental barrier against HBV infection. However, it was reported that HBV could infect the placental cells and not induce apoptosis, leading to HBV persistence in trophoblasts [142]. Its presence is often in low concentrations in the trophoblast plasma, and tumor necrosis factor-alpha (TNF-α) might enhance HBV replication in these cells [143]. In concordance with the above findings, clinical studies reported that intrauterine HBV infection occurred in approximately 3.7% of infants born to HBsAg-positive pregnant women [101,142]. Consequently, the principal mode of MTCT of HBV is thought to be during the intrapartum due to the rupture of the placental barrier during this period.

### 3.5. HSV

The infection caused by HSV is one of the most common sexually transmitted viral diseases among women of reproductive age [144]. The causative agents, HSV viruses, are enveloped double-stranded DNA viruses. HSV-1 is often discovered orally, while HSV-2 is more commonly found in genital tracts. Genital HSV infection in pregnancy was reported to be associated with spontaneous abortion, preterm labor, intrauterine growth retardation, and congenital and neonatal infections [145]. Pregnant women with primary infection may suffer from severe illness. They may likely transmit the virus to their fetus or babies, especially if the infection occurs in the latter half of pregnancy [146,147]. In addition, it is noted that primary maternal infection during the third trimester has the highest percentage of neonatal infection, and neonatal infection occurs when the fetus passes through the infected birth canal [102]. It was proved that the neonatal infection risk was reduced by caesarean section in recurrent maternal HSV infection with clinical symptoms [148,149]. It has been well established that the virus can be transmitted to the fetus in utero and cause congenital malformations such as microcephaly, microphthalmia, or hydranencephaly [150,151]. Although the syncytiotrophoblast layer is considered a barrier to maternal–fetal transmission of HSV in some studies, human trophoblasts were shown to be infected with HSVs, with the complete replicative cycle of these viruses observed in other reports, suggesting that the trophoblast layer may be involved in the mechanisms of this intrauterine HSV infection [152,153,154].

### 3.6. IAV

Of the three principal types of influenza viruses, influenza A and B viruses can be endemic, while only IAV is the cause of the worldwide influenza pandemic. Based on the two surface proteins, hemagglutinin (H) and neuraminidase (N), IAVs have been further classified. Pregnant women have been well-known for having increased risks for infection with both seasonal and pandemic IAV and influenza complications during the seasonal influenza periods [6,72,155,156,157]. Various adverse pregnancy outcomes such as spontaneous abortion, preterm birth, and death for pregnant women infected with IAV, were reported during the well-known influenza pandemic in 1918 [158,159] and the H1N1 influenza pandemic in 2009 [73,160,161]. In addition, influenza infection in pregnancy also might cause a slight increase in congenital deformities, but this was not consistently reported across studies [155,162]. Using a mouse model, Littauer et al. (2017)’s findings suggested that the disruption of tissue-specific hormonal regulation resulting from H1N1 IAV infection leads to preterm labor, impairment of fetal growth, increased morbidity and mortality, and maternal mortality [72]. Although rare occurrence or no viremia has often been mentioned in influenza [156,163], some studies employing highly sensitive PCR suggested transient viremia before the onset of respiratory infection is common [164,165]. In vitro studies reported that trophoblast cells were susceptible to IAV, both with H1N1 and H3N2 viruses, especially with the H3N2 virus, which could successfully replicate and induce apoptosis in the immortalized human first-trimester trophoblast cells [166,167]. However, vertical transmission of IAV appears to be rare, with no placental transmission noted in a clinical study in the second and third trimesters and in an animal model study using gilts [107,156].

### 3.7. ZIKV

ZIKV is a *Flavivirus*, a causative viral agent of a recently known CZS. This mosquito-borne infection was first noted in Yap Island in 2007, later in French Polynesia, and recently in Brazil and other parts of the Americas [168,169,170,171,172]. The infection usually presents with a mild fever, rashes, and joint pain. However, infection in pregnant women often results in severe medical and public health consequences and is likely to cause CRS, especially if the infections occur in the first trimester. The unfavorable outcomes for the fetus include microcephaly and other neurological birth defects, neurological disorders such as Guillain–Barre syndrome, or mental retardation for the fetus [10,173,174]. No vaccine for preventing ZIKV infection is available, and its development is still in progress [175,176,177,178].

Vertical transmission of ZIKV has been confirmed [76]. The virus can infect various cell types at the maternal–fetal interface, such as primary human placental cells, explants-cytotrophoblasts, endothelial cells, fibroblasts, and Hofbauer cells in chorionic villi. Maternal decidual tissues, amniotic epithelial cells, and trophoblast progenitors of amniochorionic membranes are also permissive for this virus [11,108,179,180,181,182].

### 3.8. SARS-CoV-2

There have been several concerns regarding the pregnancy outcomes if pregnant women are infected with SARS-CoV-2, the virus causing the current pandemic coronavirus disease of 2019 (COVID-19). Although the clinical manifestations of the infected pregnant women were not different from those of the non-pregnant, severe complications for both the mother, including preeclampsia development, and the fetus were noted for SARS-CoV-2 infection in pregnancy [46,85,183,184].

It has been reported that the vertical transmission of SARS-CoV-2 is unlikely; however, its successful transmission was noted in some cases [79,84,110,185,186,187,188]. The vertical transmission rate was estimated to be less than 3.2% [83,189,190]. Regarding mechanisms for the viral entry into susceptible cells, ACE2 is well known as a receptor for this virus. This protein is wildly expressed from the cells present at the maternal–fetal interface [191], implicating that these cells have a high chance of being infected. However, in general, the placenta barrier again works effectively to protect the fetus from this virus, and the majority of the babies born to these mothers were free from this virus infection. Clinical and in vitro evidence have demonstrated that although the placenta showed signs of infections in the invading trophoblasts and placenta, the infection seems not to go further [7,192,193]. The evidence of restricted replication of the virus in trophoblast cells was noted, suggesting that the placental barrier may be present, although not effectively, and the underlying mechanisms remain unclear [7,8].

In summary, during pregnancy, the mothers can be infected with various viral pathogens, through air-borne transmission (RuV, IAV, and SARS-CoV-2), through body fluids in sexual contact (HIV, HBV, and HSV), or in blood-borne transmission (HIV, HBV). Pregnant women can also be infected via direct contact with HSV- or CMV-infected bodily fluids, or by mosquito-borne transmission of ZIKV. Pregnant women infected with one of the above viruses can suffer from increased adverse outcomes such as miscarriage, stillbirth, or premature delivery. RuV, HCMV, and ZIKV are well known for crossing the placenta, especially in the first trimester (RuV and ZIKV), and causing congenital viral syndromes with various birth defects including the well-known microcephaly for ZIKV infection and others such as sensory loss and mental retardation. Vertical transmission during the peripartum period as well as through breastfeeding is prevalent with HIV, HBV, and HSV; especially in HBV-infected pregnant women with a high viral load in the third trimester. These two neonatal infections likely lead to liver chronic infection for HBV, or in immunocompromised status leading to AIDS if no antiretroviral therapy is received as seen in HIV. Primary HSV maternal infection during the third trimester has the highest percentage of neonatal infection, and neonatal infection occurs when the fetus passes through the infected birth canal. Among the two remaining airborne viruses, although IVA can infect trophoblasts in in vitro, a low vertical transmission rate was noted compared to other viruses such as RuV, HCMV, and ZIKV. Signs of infected placenta as well as trophoblast cells were noted with SARS-CoV-2; however, restriction replication of this virus was observed in in vitro. In addition, babies born to a mother infected with SARS-CoV-2 were often free of this virus. These above findings suggest that a placental barrier is present, although limited, to prevent the fetus from intrauterine infection. Of these eight viruses, vaccines against RuV, IVA, HBV, and SARS-CoV-2 have been available in clinical practice leading to improved pregnant outcomes. By the use of antiretroviral therapy with or without avoiding breastfeeding, vertical transmission of HIV and HBV is greatly reduced. HCMV infection is worldwide; however, it is yet an under-recognized infectious cause of newborn malformation. Since the introduction of the universal RuV vaccine, congenital HCMV has become predominant.

## 4. TGF-β1 and Its Essential Roles in Human Body Development and Reproductive Tract

The transforming growth factor (TGF) family, which divides into two main groups, the TGF-β family and the bone morphogenetic proteins, consists of secreted polypeptide growth factors that are involved in a variety of cellular processes such as cell growth and development, differentiation, extracellular matrix synthesis, migration, and apoptosis. The TGF-β family consists of three isoforms sharing approximately 70% sequence homology: TGF-β1, TGF-β2, and TGF-β3. They are secreted as latent complexes and require activation to bind to their receptors. TGF-β isoforms signal through a heteromeric complex of type I and type II serine/threonine kinase receptors, which initiate downstream signaling pathways involving Smad proteins [194,195,196]. TGF-β signaling is complex and plays important roles in regulating cellular processes, including wound healing, chemotaxis, and immune regulation. It is the main modulator of fibrosis upregulating collagen expression [197]. In pregnancy, TGF-β signaling plays a critical role in embryonic development and is essential for proper human fertility and reproduction [194,198,199,200,201]. Its dysregulation can lead to preterm delivery by altering tight junction expression [202,203].

Among the TGF-β family, TGF-β1, a pleiotropic growth factor secreted by many cell types, and the TGF-β receptor 1 (TβRI) serve essential roles in this family [199,204]. In pregnancy, TGF-β1 has been found abundant at the maternal–fetal interface. Immune cells, such as Treg cells, decidual macrophages, Hofbauer cells, and particularly first-trimester trophoblast cells, also secrete TGF-β1 [17,205,206,207,208,209,210]. During pregnancy, the maternal immune system is required to tolerate the presence of the fetus, which has a distinct set of antigens that can potentially trigger an immune response. TGF-β1 promotes an immunosuppressive environment at the maternal–fetal interface, allowing for successful pregnancy outcomes. It inhibits T cell activation, promotes regulatory T cell differentiation and proliferation, suppresses dendritic cell maturation, and induces apoptosis in potentially harmful immune cells. These functions collectively help to maintain the immune tolerance of the developing fetus [194,198,211,212].

Elevated concentrations of TGF-β1 in maternal plasma and placenta were noted in preeclamptic pregnancies [213,214]. TGF-β1 and TGF-β2 are the most abundant isoforms in CTB cell columns, but TGF-β1 is lower in invasive EVTs [200]. TGF-β signaling in the endometrium is active during implantation and has a pivotal role in regulating endometrial receptivity and embryo implantation. It is assumed that TGF-β is the factor that controls both apoptosis and proliferation of endometrial cells during embryo implantation. As being widely recognized as a core component of fibrosis, its potential contribution to the development of intrauterine adhesion has been mentioned [215]. Additionally, it is suggested to be involved in the pathogenesis of endometriosis by favoring the cell survival and proliferation of the ectopic endometrium [216]. Recently, more functions of TGF-β1 have been discovered, including its roles in viral infection, especially viral infection during pregnancy as mentioned earlier.

## 5. Roles of TGF β in Viral Infection at the Non-Maternal–Fetal Interface

Not only does the TGF-β1 play irreplaceable roles in human cell growth, differentiation, development, and immune regulation, but it also contributes different roles in various human viral infections. Enhanced expression or association with an elevated concentration of TGF-β1 in different viral infections have been thought or reported as a result of system or local immune response to protect against viral pathogens such as IAV, HIV, SARS-CoV-2, hepatitis E virus, chikungunya virus, Rift Valley Fever Virus, etc. [217,218,219,220,221,222,223]. However, its functions related to the viral infection and the viral life cycle have not always been well addressed, including the viruses of the typical ToRCH pathogens and other known potentially transplacental transmissions, or recently emerging viruses such as ZIKV and SARS-CoV-2.

### 5.1. RuV

Possible roles of TGF-β1 in RuV infection have been studied by our research group. It was found that in human lung cancer epithelial A549 cells, RuV infection was enhanced by TGF-β1. Although the underlying mechanisms have not been clarified, TGF-β1 induced a three- to five-fold increase in RuV binding to the treated A549 cells [20].

### 5.2. HCMV

It was noted that the secretion and activation of TGF-β1 are promoted in HCMV infection [224]. Although investigating the effect of TGF-β1 on HCMV replication and infection has been mentioned, a study indicated that HCMV-infected renal tubular epithelial cells could undergo EMT after exposure to TGF-β1, similar to uninfected renal epithelial cells but that HCMV infection by inducing active TGF-β1 may potentiate renal fibrosis, which helps to explain the clinical association between HCMV infection, TGF-β1, and adverse renal allograft outcomes [225]. Promotion of the activation of TGF-β1 in human umbilical vein endothelial cells by matrix metalloproteinase 2 (MMP-2) after the endothelial mesenchymal transition was addressed by Chen et al. (2019). Treatment with TGF-β1 on human umbilical vein endothelial cells infected with HCMV, can activate the extracellular potential TGF-β1 by activating MMP-2 [226]. In this research trend, another study reported that, under TGF-β1 treatment, HCMV and TGF-β1 promoted cell invasion and migration in glioma cells by the JNK pathway [227].

### 5.3. HIV

Elevated TGF-β1 in chronic HIV infection was often noted and suggested to contribute to immunosuppression in HIV-infected individuals [217,228,229,230,231]. It was proved that TGF-β1 could induce CXCR4 expression, a co-receptor for HIV binding, and HIV-1 entry in human monocyte-derived macrophages [24]. As an elevated TGF-β1 has often been found in patients with chronic lung diseases such as chronic obstructive pulmonary disease and asthma, its roles in HIV infection in primary differentiated human bronchial epithelial cells have recently been investigated. Chinnapaiyan et al. (2017) reported that ex vivo cultured primary bronchial epithelial cells and the bronchial brushings from human subjects which express canonical HIV receptors CD4, CCR5, and CXCR4 can be infected with HIV [232]. In addition, TGF-β1 promoting HIV latency by upregulating a transcriptional repressor BLIMP-1 (B lymphocyte-induced maturation protein-1) was further reported. These authors suggested that in patients with chronic airway diseases, TGF-β1 can elevate the HIV viral reservoir load that could further exacerbate the HIV-associated lung comorbidities [25]. Increased production of TGF-β which promotes immunosuppression was noted in HIV and also in simian immunodeficiency virus (SIV) infection. An enhanced intestinal TGF-β/Smad-dependent signaling in SIV-infected rhesus macaques was reported by Boby et al. (2021) [233].

### 5.4. HBV

Like other viral infections, elevated concentration and TGF-β1 production upon HBV infection have been reported in clinical and in vitro studies [27,234,235,236,237,238]. Plasma concentration of TGF-β1 was high in patients with HBV infection, especially in the first week of acute viral B hepatitis [27,235]. Subsequently, many studies investigated the role of TGF-β1 in developing hepatocellular carcinoma and its connection with liver fibrosis. Guo et al. (2009) suggested that hepatitis B virus X protein (HBx) may facilitate liver fibrosis by promoting hepatic stellate cell proliferation and upregulating the expression of fibrosis-related molecules including the TGF-β1 [236]. Although TGF-β level is thought to be an independent factor related to the occurrence of chronic HBV infection (CHB) [239], serum TGF-β1 and IL-31 were markedly higher in HBV-related liver cirrhosis (LC) patients and correlated with the severity of HBV-LC, suggesting possible roles of the TGF-β1/IL-31 pathway in the pathogenesis of liver fibrosis during CHB [237,240]. The miR-15a/Smad-7/TGF-β pathway and the TGF-β1/miR-21-5p pathway were reported to play an important role in HBV-associated liver cancer and HBV-induced liver fibrosis, respectively [29,30]. In a recent study, liver fibrogenesis promoted by HBV infection through the TGF-β1-induced OCT4/Nanog pathway has also been demonstrated [241]. Of the rare studies investigating the roles of TGF-β1 in HBV infection and replication, a study reported that TGF-β1 does not affect HBV duplication in human hepatocellular carcinoma cells HepG2.2.15 and can inhibit the expression of HBsAg and HBeA [242]. TGF-β1 was shown to suppress HBV replication effectively, and this effect was primarily through transcriptional inhibition of pregenomic RNA. The authors suggested that TGF-β1 may play a dual role in HBV infection, in the suppression of immune responses against viral infection and the direct inhibition of viral replication [28].

### 5.5. HSV

Early in this century, induction of the release of TGF-β1 protein was noted in in vitro infection of human mononuclear cells with HSV type 1 (HSV-1). This TGF-β1 production was highly significant after 48 h [243]. High concentrations of TGF-β1 in peripheral blood was also confirmed in patients infected with HSV-1 or HCMV and other viruses such as Varicella-zoster virus, Epstein–Barr virus, and mumps virus [244]. However, the above increased TGF-β1 production seems to be cell type-dependent, as its suppressive expression was found in HSV-1-infected human corneal epithelial cells and HSV-1-infected human trabecular meshwork cells [245,246]. On the other hand, TGF-β signaling results in increased HSV-1 latency in a mouse model [22]. TGF-β1 exposure enhances HSV-1 replication along with a significant reduction in CXCL10 expression in 3-dimensional human corneal keratocyte cultures [247].

### 5.6. IAV

An elevated concentration of TGF-β1 was noted in severe cases of influenza A H1N1 infection in an early report [248]. A reciprocal TGF-β1–integrin crosstalk regulated by the immune adapter ADAP (Adhesion and Degranulation-promoting Adapter Protein) is suggested to play a protective role against influenza infection [249]. An increase in TGF-β1 was also noted in nasal mucosal lining fluid collected from neonates of mothers receiving A (H1N1) pnd09 vaccination during pregnancy [250]. In the mucosal immune response, the role of epithelial-derived TGF-β1 in suppressing early interferon β responses leading to increased viral burden and pathology was noted [21]. On the other hand, pre-treatment of TGF-β1 significantly inhibited apoptosis and the presence of proapoptotic factors of H1N1-infected A549 cells in an in vitro study [251].

### 5.7. ZIKV

There has been a limited number of published articles pertaining to this review topic. It has been suggested that TGF-β1 may play a role in the immune response and pathogenesis of ZIKV infection. ZIKV infection induced increased expression of TGF-β along with other proinflammatory and anti-inflammatory cytokines in the neural parenchyma in fetal cases of microcephaly [252]. In recent reports, TGF-β1 does not affect the replication of ZIKV in Setoli cells in an in vitro study using a multiplicity of infection (MOI) of one [253]. However, as mentioned earlier, it does increase ZIKV replication in the first-trimester trophoblast cells at the maternal–fetal interface.

### 5.8. SARS-CoV-2

In the current COVID-19 pandemic, which has caused many severe death cases for people worldwide since the beginning, changes in TGF-β1 expression at SARS-CoV-2 targeted tissues and its concentration in serum samples have been investigated. Studies have reported a low mRNA expression of TGF-β1 at the mRNA level in the early inflammatory response in upper airway samples [33] or no elevated concentration of TGF-β1 in blood samples at diagnosis of COVID-19 by PCR suggesting no help to anticipate long-term prognosis [254]. However, upregulation of TGF-β1 was often reported, especially in COVID-19 cases associated with lung injury. Serum levels of TGF-β1 were significantly increased at the early and middle stages of COVID-19 and correlated with the levels of SARS-CoV-2-specific IgA [32]. In an immunohistochemical analysis study, using paraffin lung samples from patients who died of COVID-19, a significant increase in the immunoexpression of TGF-β1 was observed compared to control groups. Recently, Laloglu and Alay (2022) reported that in patients with confirmed COVID-19 and pulmonary involvement, along with elevated connective tissue growth factor levels, significantly high concentrations of TGF-β1 in serum samples were noted, especially in more severe pneumonia groups [255]. The authors suggested that TGF-β1 is one of the potential markers that can distinguish COVID-19 patients with pulmonary involvement and indicate disease severity. TGF-β1 has been suggested to play some crucial roles in SARS-CoV-2 infection. Subsequently, the use of some TGF-β1 inhibitors has been proposed to mitigate the current COVID-19 pandemic [31,256].

The reported possible roles of TGF-β1 in various viral infections including those mentioned above, have been summarized in Table 3 and Table 4.

## 6. TGF-β1 and Viral Infection at the Maternal–Fetal Interface

**Figure 2:**
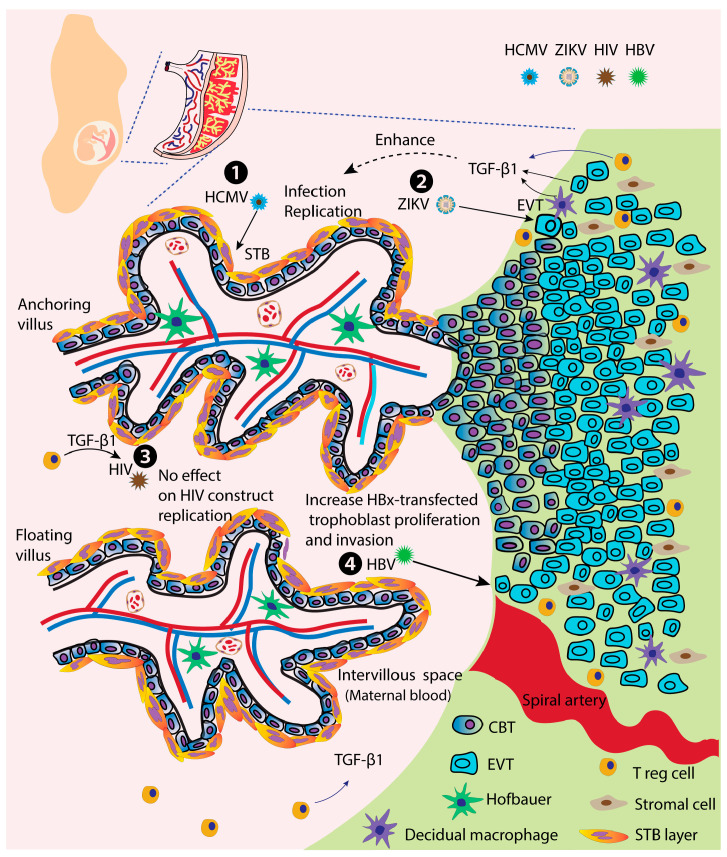
Trophoblasts, microvillous structure and the published effects of TGF-β1 on viral infection in trophoblasts at the maternal–fetal interface. ❶, TGF-β1 increases the infection and replication of HCMV in STBs through macrophage contact [26]; ❷, enhancement of ZIKV infection and replication in trophoblasts by TGF-β1 [34]; ❸, no effect on HIV construct replication in trophoblasts under TGF-β1 treatment [257]; ❹, increases HBx-transfected trophoblast proliferation and invasion [258]. Abbreviations: HCMV, human cytomegalovirus; HBV, hepatitis B virus; HIV, human immunodeficiency virus; ZIKV, Zika virus; CBT, cytotrophoblast; STB, syncytiotrophoblast; EVT, extravillous trophoblast; T reg, regulatory T cells.

Although there have been several studies on the topic of TGF-β1 and viral infection as mentioned above, reports addressing the possible roles of TGF-β1 in viral infection at the maternal–fetal interface are quite limited. Quite a few published studies conducted in in vitro with relevant contents reported the TGF-β1 roles in viral infection in trophoblast cells, the critical barrier in protecting the fetus from maternal viral infection (Figure 2 and Table 4).

### 6.1. HCMV

At the end of the last century, Bacsi et al. (1999) reported a necessary contact of placental macrophage for HCMV replication in STBs [26]. TGF-β1 and interleukin-8 (IL-8), which are released from placental macrophages, promote the complete replicative cycle of HCMV in the studied STBs. The findings indicated an interactive role for the STB layer and placental macrophages in the dissemination of HCMV among placental tissue, contributing to the transmission of HCMV from the mother to the fetus. On the other hand, a recent study using EVTs isolated from early placentae reported an elevated level of TGF-β1 protein in HCMV-infected EVT cells at 48 h postinfection, suggesting a role of HCMV in the proliferation and invasion of these EVT cells [259].

### 6.2. HIV

Early in this century, Zachar et al. (2002) tested the roles of various cytokines and growth factors (including TGF-β1) typically present in the placental micro-environment in HIV infection in trophoblasts. Unlike the following four cytokines, epidermal growth factor (EGF), granulocyte-macrophage colony-stimulating factor (GM-CSF), IL-1β, and TNF-α, which showed stimulation of promoters of tested HIV viruses, no effect on the transcriptional expression of the promoter constructs was noted with the TGF-β1 [257].

### 6.3. HBV

In an in vitro model, Cui et al. (2015) induced a multifunctional viral regulator of HBV gene products, HBx, and its different fragments to overexpress in a trophoblast cell line, HTR-8/SVneo. The authors reported that TGF-β1 decreases HTR-8/SVneo cell proliferation and invasion while increasing HBx-transfected HTR-8/SVneo cell proliferation and invasion [258].

### 6.4. ZIKV

Recently, our research group presented an in vitro study testing possible roles of TGF-β1 in ZIKV infection in the immortalized human first-trimester trophoblast cells Swan. 71. By using enough MOI to assure every single cell has a chance to come in contact with at least one virus particle theoretically. The results showed an enhancement in ZIKV binding and replication in these trophoblast cells. In addition, such enhancement effects were abolished using an inhibitor of the Smad pathway, SB431542 or SB525334 [34].

## 7. The Smad Pathway and Promising Future Approaches

**Figure 3:**
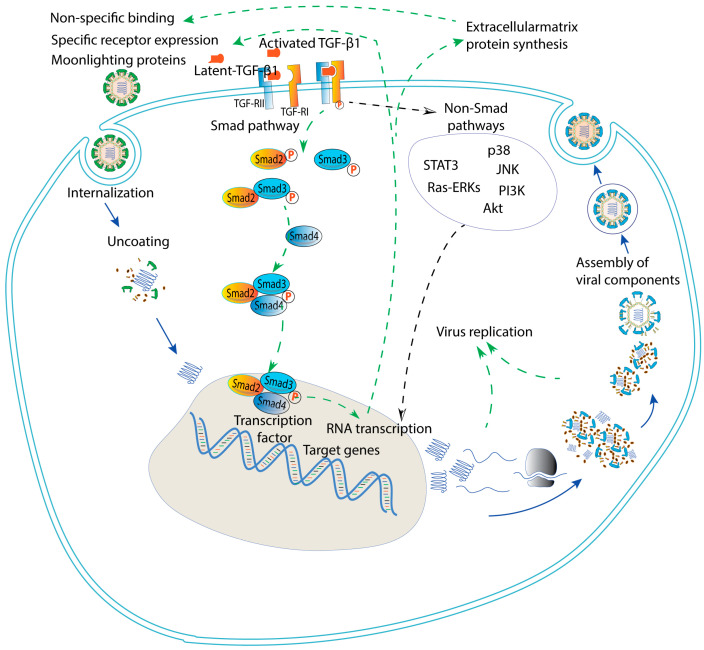
The Smad pathway and possible underlying mechanisms of the published effects of TGF-β1 on ZIKV infection in relation to the virus life cycle.

One of the major downstream signaling pathways of TGF-β1 is the Smad pathway. Smads are intracellular signaling proteins that transduce TGF-β signaling from the cell surface to the nucleus, where they regulate gene expression. The Smad pathway is initiated by activating and binding TGF-β1 to its receptors, TβRI and TβRII, resulting in the phosphorylation of receptor-regulated Smad2/3 proteins [260]. This forms complexes with other Smad proteins (Smad4) and translocate to the nucleus where they regulate gene transcription in a cell-specific manner (Figure 3).

To date, besides studies reporting the elevated concentration or an enhancement of TGF-β1 as a systemic response or locally from infected tissues, several studies investigated the roles of TGF-β1 in viral infection and replication. It has been shown that TGF-β1 could promote the infection and replication of some viruses as mentioned earlier, and in some cases their underlying mechanisms are still being investigated. It is thought that TGF-β1 might enhance the non-specific binding of a given virus to extracellular matrix proteins, which play a cofactor supporting the viral entry. There is also a possibility that TGF-β1 promotes viral infection via effects on the expression of moonlighting proteins. A moonlighting protein is a protein that has multiple functions in addition to its primary role. Heat-shock proteins (HSP) such as HSP90, glucose-regulated protein 94 (GRP94), and GRP78 may have other functions such as signal transduction, immunoregulation, and especially, working as cellular receptors for some viruses, in addition to their primary roles as chaperone proteins in assisting in protein folding and refolding within the endoplasmic reticulum [261,262,263,264,265,266,267,268]. Other moonlighting proteins, such as annexin A2 and cyclophilin A, have been shown to play roles in the entry of several viruses, including HIV, IAV, and SARS-CoV-2 [269,270,271,272,273]. These proteins can interact with the viral envelope proteins and serve as cellular cofactors supporting the viral entry. Further studies are necessary to clarify these mechanisms in viral infection during pregnancy.

It is noteworthy that TGF-β1 could induce enhanced expression of cellular receptors for viral entry via the Smad pathway. In recent SARS-CoV-2 research, Mezger and colleagues reported that activation of the Smad pathway via TGF-β1 or ALK5 agonists led to increased expression of furin, a protease that cleaves the spike protein of SARS-CoV-2, in a broad spectrum of cells including Huh-7 (a permanent cell line established from male hepatoma tissue), and Calu-3 cells (epithelial cells isolated from lung tissue derived from a male patient with lung adenocarcinoma), which enhanced susceptibility to SARS-CoV-2 infection in these cells [256]. Expanding the above findings to further studies to investigate possible mechanisms of transplacental infection of the SARS-CoV-2 is highly recommended.

One of the reported studies addressing the role of TGF-β1 in viral infection in pregnancy is our recent work on Zika virus infection in trophoblast cells with the predominant role of the Smad pathway. The enhancement effect of ZIKV induced by TGF-β1 might be attributed to an increase in cellular receptors of ZIKV, AXL and Tyro3 [34]. In addition, increasing extracellular matrix synthesis resulting from the Smad pathway leading to an enhancement of non-specific binding of ZIKV to the trophoblast cells may not be excluded. Studying possible activated or moonlighting proteins as downstream results of the Smad pathway turning into cellular receptors for ZIKV can be paid more attention to. Possible downstream factors of the Smad pathway leading to an increase in ZIKV replication in trophoblast cells should also be investigated. Importantly, the findings on the effects of TGF-β1 in viral infection and replication were extracted from in vitro experiments, which may differ from those of actual intrauterine in vivo experiments. Therefore, expanding the experiments to other trophoblast cells, ex vivo using explant cultures or mouse model, is indeed necessary. Lastly, it has been known that, in pregnancy, a complex cytokine network is present at the maternal–fetal interface to support normal growth and development of the placenta and fetus. Therefore, not only the TGF-β1 but the possible roles of other cytokines and factors present at the maternal–fetal interface should also be studied.

## Figures and Tables

**Table 1:** Summary of general information related to the reviewed viral infections.

Virus	Transmission Routes	Major Infected Cells/Organs	Pregnant and Fetal Outcomes	Vaccine Availability	Representative References
RuV	Respiratory tract. Direct or droplet contact	Respiratory mucosa and cervical lymph nodes. Others: skin, eye, brain	CRS	Yes	[48,49,50,51,52]
HCMV	Through bodily fluids: saliva, urine, blood, breast milk	Epithelial cells, fibroblasts, endothelial cells, and immune cells	Congenital CMV infection	No	[53,54,55,56]
HIV	Sexual contact, sharing injecting equipment	Immune system, primarily targeting CD4+ T cells	Increase miscarriage, stillbirth, or premature delivery. Congenital HIV infection	No	[57,58,59,60,61]
HBV	Sexual contact, sharing injecting equipment	Liver cells	Premature delivery or low birth weight, chronic HBV	Yes	[62,63,64,65]
HSV	Sexual contact, infected skin or mucous membranes	Skin and mucous membranes, nerve cells	Congenital HSV infection, lead to neurological damage, blindness, and death	No	[66,67,68,69]
IAV	Respiratory tract, through respiratory droplets	Primarily infects respiratory tract cells	Increased risk of pneumonia, premature delivery, or stillbirth.	Yes	[6,45,70,71,72,73]
ZIKV	Aedes mosquito bite, sexual contact, blood transfusion	Infects skin, lymph nodes, and other tissues including placenta	Fetal loss, stillbirth, miscarriage, CZS with brain abnormalities	No	[74,75,76,77,78]
SARS-CoV-2	Respiratory droplets	Primarily infects cells in the respiratory tract	Preterm delivery, fetal distress, and stillbirth	Yes	[46,79,80,81,82,83,84,85]

**Table 2:** Primary routes of vertical transmission of the eight viruses.

Virus	Main Routes of Vertical Transmission	Representative References
RuV	The virus can cross the placenta. Transplacental infection can occur at any stage of pregnancy, highest incidence during the first trimester (organogenesis period)	[9,92,93]
HCMV	Placental and perinatal transmissions, especially if the mother has a primary infection during pregnancy or at the time of delivery; through breastfeeding	[55,93,94,95]
HIV	The majority of MTCT of HIV occurs during delivery or through breastfeeding	[57,58,96,97,98]
HBV	Perinatal transmission during delivery is the primary route	[62,99,100,101]
HSV	Any stage of pregnancy, highest during delivery when the fetus passes through the infected birth canal	[68,102,103,104,105]
IAV	Through respiratory secretions. The risk of vertical transmission is low compared to other viruses	[3,45,106,107]
ZIKV	Can cross the placenta. Vertically transplacental infection is highest during the first and second trimesters of pregnancy	[75,76,90,108]
SARS-CoV-2	Risk of vertical transmission is generally low. Higher in certain situations: severe maternal COVID-19, infected close to the time of delivery	[79,83,109,110]

**Table 3:** Summary of findings from up-to-date reported studies on the roles of TGF-β1 in viral infections at non-maternal–fetal interface.

Virus	Type of Studies, Involved Organs/Cell Types	Effects/Roles	Reference
RuV	In vitro, lung epithelial cells	Increase the virus binding and infection in A549 cells	[20]
HCMV	In vitro, renal tubular epithelial cells and umbilical vein endothelial cells	Increased expression and activation of TGF-β1 by HCMV infection	[225,226]
HIV	Ex vivo, bronchial epithelial cells. In vitro, macrophages	Increase CXCR4 expression in macrophages, increase the viral burden in bronchial epithelial cells	[24,25,232]
HBV	In vitro, hepatocellular carcinoma cells	Inhibit the expression of HBsAg and HBeAg, and suppress HBV replication in HepG2 cells	[28,30]
HSV	Ex vivo, human cornea organotypic culture	Enhance HSV-1 replication in 3-dimensional human corneal keratocytes	[247]
IAV	In vitro, lung epithelial cells	Inhibit apoptosis induced by IAV infection on A549 cells	[251]
ZIKV	In vitro, Sertoli cells	Not affect ZIKV replication in human Sertoli cells	[253]
SARS-CoV-2	In vitro, airway epithelial cells	Increase furin expression leading to enhanced susceptibility to SARS-CoV-2	[31,256]

**Table 4:** Summary of findings from up-to-date reported studies on the roles of TGF-β1 in viral infections during pregnancy. Abbreviation: NR, not reported.

Virus	Type of Studies, Organs/Cells Involved, Pregnancy Period	Effects/Roles	Reference
RuV	NR	NR	
HCMV	In vitro, STB	TGF-β1 and IL-8 promote HCMV replication in STB	[26]
HIV	In vitro, STB	Not increase HIV construct replication	[257]
HBV	In vitro, first-trimester trophoblast HTR-8/SVneo cells	Increases HBx-transfected HTR-8/SVneo cell proliferation and invasion	[258]
HSV	NR	NR	
IAV	NR	NR	
ZIKV	In vitro, first-trimester tropho-blast cells	Increase the virus binding and replication in trophoblasts	[34]
SARS-CoV-2	NR	NR	

## Data Availability

Not applicable.

## Abbreviations

TGF-β1	Transforming growth factor-beta 1
CRS	Congenital rubella syndrome
CZS	Congenital Zika syndrome
MTCT	Mother-to-child transmission
RuV	Rubella virus
HCMV	Human cytomegalovirus
HIV	Human immunodeficiency virus
HBV	Hepatitis B virus
HSV	Herpes simplex virus
IAV	Influenza A virus
ZIKV	Zika virus
SARS-CoV-2	Severe acute respiratory syndrome coronavirus 2
SIV	Simian immunodeficiency virus
STB	Syncytiotrophoblasts
CTB	Cytotrophoblasts
EVT	Extravillous trophoblasts
MOI	Multiplicity of infection
HBx	Hepatitis B virus X protein
HSP	Heat-shock proteins
GRP	Glucose-regulated protein
COVID-19	Coronavirus disease of 2019
ToRCH	Toxoplasma, others, rubella, cytomegalovirus, herpes simplex virus
